# Effectiveness of the 2010 and 2011 Southern Hemisphere trivalent inactivated influenza vaccines against hospitalization with influenza-associated acute respiratory infection among Thai adults aged ≥50 years

**DOI:** 10.1111/irv.12233

**Published:** 2014-02-03

**Authors:** Fatimah S Dawood, Prabda Prapasiri, Peera Areerat, Asadang Ruayajin, Malinee Chittaganpitch, Charung Muangchana, Henry C Baggett, Sonja J Olsen

**Affiliations:** a Influenza Division, Centers for Disease Control and PreventionAtlanta, GA, USA; b Provincial Health OfficeNakhon Phanom, Thailand; c Provincial Health OfficeSa Kaeo, Thailand; d National Institute of Health, Thailand Ministry of Public HealthBangkok, Thailand; e Ministry of Public Health, National Vaccine Committee OfficeBangkok, Thailand; f International Emerging Infections Program, Global Disease Detection Center, Thailand MOPH-U.S. CDCNonthaburi, Thailand

**Keywords:** Case–control studies, elderly, flu vaccines, hospitalization, influenza vaccine

## Abstract

**Background:**

Inactivated influenza vaccine (IIV) effectiveness has been evaluated among older adults in high-income countries, but data on IIV effectiveness in low- and middle-income countries remain sparse. We conducted a test-negative case–control analysis to estimate 2010 and 2011 trivalent IIV effectiveness against hospitalization with influenza-associated acute respiratory infection (ARI) among persons aged ≥50 years in rural Thailand.

**Methods:**

During 2010–2011, active surveillance for ARI hospitalization was conducted in two provinces; patients were tested for influenza viruses by real-time RT-PCR. Vaccination status was obtained from vaccine registries. Case and control patients were patients with nasopharyngeal swabs positive and negative for influenza viruses, respectively. Vaccine effectiveness (VE) was estimated for the 6 months after vaccination began. Logistic regression was used to evaluate the association between case status and vaccination while adjusting for age, province, medical conditions, and time.

**Results:**

During 2010–2011, there were 1545 patients with ARI, of whom 279 (18%) were influenza-positive case patients and 1266 (82%) were influenza-negative control patients. Of the 279 case patients, 247 (89%) had influenza A and 32 (11%) had influenza B. Fourteen of 279 (5%) case patients and 108 of 1266 (9%) control patients were vaccinated against influenza. The unadjusted IIV effectiveness against hospitalization with influenza-associated ARI was 43% (95% CI: 0–68%); adjusted VE was 47% (95% CI: 5–71%).

**Conclusion:**

The 2010 and 2011 IIVs were moderately effective against hospitalization with influenza-associated ARI among Thais aged ≥50 years, but IIV coverage was low. Additional efforts are warranted in Thailand to improve IIV uptake in this target group.

## Introduction

Adults aged ≥65 years are at increased risk for influenza-associated hospitalization and death.^1^–^3^ Influenza vaccination is the most effective method of influenza prevention. Inactivated influenza vaccine (IIV) effectiveness against laboratory-confirmed influenza has been evaluated among older adults in high-income countries, some of which have long-standing recommendations for influenza vaccination for this age group.^4^–^6^ However, data on influenza vaccine effectiveness (VE) in low- and middle-income countries remain sparse. As more low- and middle-income countries consider influenza vaccination recommendations for older adults, studies are needed to evaluate influenza VE in these settings where VE may differ due to differences in the underlying health and nutritional status of the population. Additionally, influenza VE estimates in these countries are critical to assessing the impact of newly implemented influenza vaccination recommendations.

Randomized controlled trials are considered the gold standard for evaluating influenza vaccine efficacy but are expensive and difficult to perform in populations for which influenza vaccines are already recommended. A test-negative case–control design using data from systematic surveillance for respiratory illness has been used to estimate influenza VE in high-income countries.^7^–^12^ In this approach, influenza vaccination is evaluated as the exposure of interest, and case patients and test-negative control patients are defined as persons with respiratory illness with and without laboratory-confirmed influenza, respectively. This approach could also be used in low- and middle-income countries with influenza vaccination programs and systematic surveillance for respiratory illness in a large population.

Thailand is a middle-income country with a high burden of influenza in older adults. During 2005 through 2008, it was estimated that influenza resulted in 2000–12 000 pneumonia hospitalizations and 30–500 associated deaths annually among persons aged ≥50 years in Thailand.^2^ Influenza virus circulation was found to peak during June–October with a second peak sometimes occurring in January–April.^2^ In 2008, the Thailand Ministry of Public Health (MOPH) recommended influenza vaccination of persons aged ≥65 years with one or more medical conditions. In 2009, the MOPH expanded the recommendation to include all persons aged ≥65 years and all persons with one or more medical conditions. During 2010–2011, the strain composition of the Southern Hemisphere IIVs was the same and matched the 2009–2010 and 2010–2011 Northern Hemisphere formulations. The A/California/7/2009 (H1N1), A/Perth/16/2009 (H3N2), and B/Brisbane/60/2008 strains in the vaccines were also antigenically similar to influenza A/H1, A/H3, and B viruses that co-circulated in Thailand during 2010–2011. We conducted a test-negative case–control analysis using data from population-based surveillance for hospitalizations with acute respiratory tract infection (ARI) and vaccine registries to evaluate the effectiveness of the 2010 and 2011 trivalent IIVs against hospitalization with influenza-associated ARI among persons aged ≥50 years in rural Thailand.

## Methods

### Setting

Sa Kaeo (pop 526 432) and Nakhon Phanom (pop 734 000) are rural provinces located in southeast and northeast Thailand, respectively. In 2010, there were approximately 327 000 persons aged ≥50 years residing in both provinces.^13^ The proportion of the population aged ≥50 years in the two provinces is similar to the rest of Thailand (22% in Sa Kaeo and Nakhon Phanom versus 23% in Thailand). However, the *per capita* gross domestic product of Sa Kaeo and Nakhon Phanom ranks in the bottom half of all 76 Thai provinces (Sa Kaeo 51st and Nakhon Phanom 72nd).

In Thailand, Southern Hemisphere influenza vaccine is allocated annually by the Thai government to provincial health offices based on the number of persons in three vaccine target groups (healthcare personnel, persons aged ≥65 years, and persons with underlying disease) residing in each province. Influenza vaccine is then provided free of charge to persons in recommended target groups through district and provincial clinics throughout each province. Each province maintains vaccine registries to track influenza vaccine delivery and uptake.

### Data sources

During 2010–2011, active, population-based surveillance for hospitalization with ARI was conducted in all 20 hospitals in Sa Kaeo and Nakhon Phanom as previously described.^2^ ARI was defined as an acute infection (either reported fever, reported chills, measured temperature >38·2 or <35°C, or an abnormal white blood cell count or differential) and signs of lower respiratory tract disease (abnormal breath sounds, documented tachypnea, or observed cough, sputum production, or dyspnea). Patients with ARI were prospectively identified, and every other patient with ARI was systematically sampled for potential enrollment in a respiratory pathogen study. Those who consented to participation had a nasopharyngeal swab specimen collected within 72 hours of hospitalization for real-time reverse transcription polymerase chain reaction testing for respiratory syncytial virus, adenovirus, parainfluenza viruses, human metapneumovirus, and influenza viruses A and B. Data were collected on whether patients had cancer and cardiovascular, renal, cerebrovascular, and liver diseases. Of patients sampled for potential enrollment in the respiratory pathogen study, approximately half consented to enrollment during the study period. As previously described, patients who enrolled in the study were less likely to have respiratory failure requiring mechanical ventilation and to die compared to patients who did not enroll, suggesting that the study sample was biased toward patients with less severe respiratory illness.^14^

Individual influenza vaccination records from provincial vaccine registries were linked to patient records from the respiratory pathogen study using name, address, and age. Linkage was performed by the chief provincial officers who checked for mismatches due to spelling inaccuracies. All patient identifying information was removed from the resulting dataset prior to analysis by the other co-investigators.

### Data analysis

Case patients and test-negative control patients were selected from among patients aged ≥50 years enrolled in the respiratory pathogen study during July–December of 2010 and 2011. The period of analysis was selected based on a 6-month window after the first month of influenza vaccination each year in the study provinces; this period also included the peak influenza circulation months (defined as those with >10 patients hospitalized with influenza during the month). Case patients were persons hospitalized with ARI with nasopharyngeal swabs positive for influenza viruses; test-negative control patients were persons with nasopharyngeal swabs negative for influenza viruses. Influenza vaccination was defined as receipt of trivalent IIV ≥14 days and ≤182 days prior to hospitalization to limit the effect of waning immunity. Persons receiving monovalent influenza A(H1N1)pdm09 IIV were excluded from the analysis (*n* = 24).

Chi-squared test was used to compare categorical variables in bivariate analysis. The Mann–Whitney test was used to compare continuous variables. Logistic regression was used to evaluate the association between case patient and test-negative control patient status and receipt of influenza vaccination in multivariate analysis while adjusting for sex, age, province of residence, presence of ≥1 underlying medical conditions, smoking status, and timing of hospital admission (using either peak influenza months versus non-peak months or month and year of admission). Influenza VE was calculated as (1 − Odds ratio) × 100 for pooled data from 2010 and 2011; VE estimates stratified by year were also calculated as a sensitivity analysis. All analyses were performed with SAS version 9.1 (SAS Institute Inc., Cary, NC).

### Sample size calculations

The only published randomized controlled trial of IIV among persons aged ≥65 years which was conducted in the Netherlands estimated that the 1991–1992 Northern Hemisphere IIV was 58% effective in preventing laboratory-confirmed influenza.^4^ Assuming a vaccination coverage of 10% among control patients, a type I error probability of 5% and a type II error probability of 20% (power 80%), we estimated *a priori* that 380 case patients and 770 control patients would be needed for an unmatched 1:3 case–control design to detect an unadjusted VE of 50%.

### Human subjects review

The respiratory pathogen study was approved by the ethical review committees of the Thailand MOPH and the U.S. Centers for Disease Control and Prevention (protocol number 3754). This analysis was determined to be a non-research program evaluation of Thailand's national influenza vaccination program and was exempt from additional ethical committee review.

## Results

**Figure 1:**
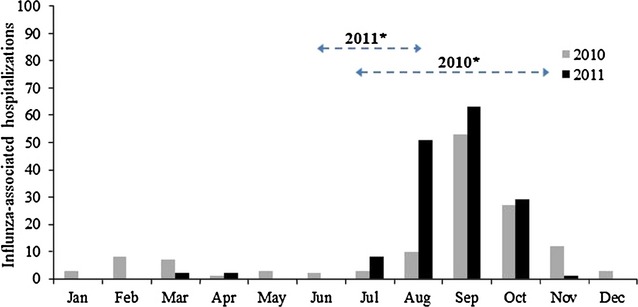
Influenza-associated acute lower respiratory tract illness hospitalizations among persons aged ≥50 years by month, 2010 and 2011, (*N* = 1545). *Horizontal dashed bars indicate period when ≥10 patients per month reported receiving trivalent IIV (i.e., the period when influenza vaccine was given in study communities).

During 2010 and 2011, 3059 patients hospitalized with ARI were enrolled into the respiratory pathogen study. Influenza viruses were detected in at least 10 patients per month during August–November 2010 and August–October 2011, which were defined as periods of peak influenza virus detection (Figure1). At least 10 patients received trivalent IIV each month during July–October 2010 and June–August 2011, indicating the period of vaccine delivery in the study communities.

During July–December 2010 and 2011, 1545 patients with ARI were enrolled, of whom 279 (18%) were influenza virus-positive case patients and 1266 (82%) were influenza virus-negative control patients; 279 (22%) of the 1266 influenza virus-negative control patients were positive for adenovirus, RSV, parainfluenza viruses or hMPV. Of the 279 case patients, 247 (89%) had influenza A and 32 (11%) had influenza B. Case patients and control patients did not differ in age or presence of ≥1 underlying medical conditions, but a larger proportion of case patients were from Nakhon Phanom than Sa Kaeo (63% versus 37%, *P* < 0·01) (Table1). The median length of hospitalization of case patients was 4 days (IQR 4–6), 4% had respiratory failure, and 2% died during hospitalization. Fourteen of 279 (5%) case patients and 108 of 1266 (9%) control patients were vaccinated against influenza. Vaccinated and unvaccinated patients did not differ in age distribution, presence of ≥1 underlying medical conditions, or province of residence (Table2). However, compared to unvaccinated patients, a larger proportion of vaccinated patients were female (48% versus 57%, *P* = 0·04) and had cardiovascular disease (22% versus 33%, *P* < 0·01), whereas a smaller proportion were smokers (27% Versus 17%, *P* = 0.02).

**Table 1:** Baseline characteristics and outcomes of patients hospitalized with acute lower respiratory tract illness stratified by case versus control status, July–December 2010 and 2011, (*N* = 1545)

	Case patients (*n* = 279)		Control patients (*n* = 1266)		*P*-value
		
	*n*	%	*n*	%	
Male	141	51	652/1265	52	0·8
Median age (IQR)	68	(59–75)	69	(60–77)	0·2
Age group (years)					
50–64	107	38	476	38	0·04
65–74	99	35	370	29	
≥75	73	26	420	33	
Current smoker	57/266	21	327/1202	27	0·05
≥1 underlying condition**	92	33	417/1255	33	0·9
Cardiovascular disease	57	20	287	23	0·4
Renal disease	33	12	115	9	0·2
Cancer	7	3	27	2	0·7
Cerebrovascular disease	10	4	25	2	0·1
Liver disease	4	1	20	2	0·8
Province					
Sa Kaeo	103	37	610	48	<0·01
Nakhon Phanom	176	63	656	52	
Vaccinated against influenza*	14	5	108	9	0·05
Median length of hospitalization (IQR)	4	(4–6)	5	(4–8)	<0·01
Respiratory failure requiring intubation	9/256	4	110/1193	9	<0·01
Death	6/255	2	45/1193	4	0·3

Denominators reflect number of patients for whom data were available when data were missing for some patients.

* Vaccinated is defined as receipt of TIV ≥14 days and ≤182 days prior to hospitalization.

** Participants were asked about the following medical conditions: liver, renal, cardiovascular and cerebrovascular disease and cancer.

**Table 2:** Baseline characteristics and outcomes of patients hospitalized with acute lower respiratory tract illness stratified by influenza vaccination status, July–December 2010 and 2011, (*N* = 1545)

	Vaccinated*(*n* = 122)		Unvaccinated (*n* = 1423)		*P*-value
		
	*n*	%	*n*	%	
Male	52	43	741/1422	52	0·04
Median age (IQR)	71	(61–76)	68	(59–77)	0·3
Age group (years)					
50–64	38	31	545	38	0·2
65–74	45	37	424	30	
≥75	39	32	454	32	
Current smoker	20/116	17	364/1352	27	0·02
≥1 underlying condition**	44/121	36	465/1413	33	0·4
Cardiovascular disease	40	33	304	22	<0·01
Renal disease	6	5	142	10	0·07
Cancer	2	2	32	2	0·7
Cerebrovascular disease	1	1	34	2	0·3
Liver disease	0	0	24	2	0·2
Province					
Sa Kaeo	61	50	652	46	0·2
Nakhon Phanom	61	50	771	54	
Influenza virus positive	14	11	265	19	0·05
Influenza A	11	9	236/1417	17	0·03
Influenza B	3	2	29/1417	2	0·8
Median length of hospitalization (IQR)	5	(3–7)	5	(4–7)	0·08
Respiratory failure requiring intubation	11/116	9	108/1333	8	0·6
Death	1/114	1	50/1333	4	0·1

Denominators reflect number of patients for whom data were available when data were missing for some patients.

* Vaccinated is defined as receipt of TIV ≥14 days and ≤182 days prior to hospitalization.

** Participants were asked about the following medical conditions: liver, renal, cardiovascular and cerebrovascular disease and cancer.

The unadjusted IIV effectiveness against hospitalization with influenza-associated ALRI was 43% (95% CI: 0–68%) (Table3). After adjusting for age group, province of residence, presence of underlying medical conditions, and timing of hospital admission, influenza VE was 47% (95% CI: 5–71%). Inclusion of sex and smoking status in the logistic regression model did not change the effect size of influenza vaccination. Therefore, sex and smoking status were excluded from the final model. Additionally, controlling for time by calendar month and year instead of peak versus non-peak period of influenza virus circulation did not alter the effect size of influenza vaccination. VE estimates stratified by year and VE estimates adjusted for calendar month and year instead of peak versus non-peak periods of influenza virus circulation are shown in Table3. Influenza VE could not be estimated by the presence of underlying condition, age, or influenza type or subtype due to the small number of case patients.

**Table 3:** Inactivated influenza vaccine effectiveness against hospitalization with influenza-associated ALRI, July–December 2010 and 2011, (*N* = 1545)

	Unadjusted VE	95% CI		Adjusted VE	95% CI	
2010 and 2011 seasons	43%	0	68	47*	5	71
Sensitivity analyses						
2010 season**	35	−7	75	17	−127	70
2011 season**	47	−7	74	52	−1	77
2010 and 2011 seasons adjusting for time as						
month and year of circulation***	–	–	–	45	0	70

* Adjusted for age, presence of ≥1 underlying medical conditions, time as peak versus non-peak influenza virus circulation, and province.

** Season defined as July–December of each year.

*** Instead of peak versus non-peak influenza virus circulation.

## Discussion

We estimated that the 2010 and 2011 Southern Hemisphere vaccines were 47% effective at preventing hospitalization with influenza-associated ARI among persons aged ≥50 years. However, reported influenza vaccine uptake was low with only 9% of control patients reporting receipt of influenza vaccine during the concurrent year. Receipt of influenza vaccination in this analysis is not a direct measure of community vaccination coverage because participants were all hospitalized and because reported vaccination reflects only vaccination received prior to hospitalization. However, the low proportion of patients reporting influenza vaccination suggests that additional efforts are needed in Thailand to improve influenza vaccine uptake in target groups. Thai influenza vaccination program efforts may also need to focus on earlier delivery of influenza vaccine to target groups, as reported influenza vaccination among participants in this analysis coincided with peak influenza virus detection during 2010.

The choice between outpatient and inpatient case patients and control patients and the resulting point estimates of influenza VE among older adults (e.g. ≥50 years or ≥60–65 years) have varied substantially in other case–control studies. In studies evaluating Northern Hemisphere 2010–2011 IIV against influenza using primarily outpatient case and control patients, point estimates of VE among persons ≥60 years ranged from no significant effectiveness likely due to small sample sizes to approximately 70% effectiveness.^8^,^11^,^12^ In test-negative case–control studies evaluating the same vaccine strain composition against influenza-associated hospitalization in persons ≥60 years, point estimates ranged from 52% in a study using hospitalized case patients and outpatient control patients^10^ to 59% in a study using hospitalized control patients,^9^ consistent with our VE estimate.

Several assumptions are made when using the test-negative case–control design to estimate influenza VE. Among these are the assumptions that healthcare-seeking behaviors, including propensity to be vaccinated, do not differ by case and control status and that cases and controls are representative of the population to which results will be generalized.^15^ We believe our study meets the first assumption because both cases and controls were hospitalized and therefore were likely to have similar access to medical care. Additionally, although data on some potential confounders such as baseline functional status were not available because data were originally collected for surveillance purposes, available data on patients' underlying conditions suggest that the underlying health statuses of cases and controls were similar. In contrast, cases and controls are probably not representative of the general population of persons aged ≥50 years in the study provinces because persons who require hospitalization for ARI are likely to have poorer underlying health statuses which may influence response to influenza vaccination. Therefore, our VE estimate may underestimate IIV effectiveness for the general population of persons aged ≥50 years, but reflects VE in the most vulnerable segment of the population that is targeted for influenza prevention measures in Thailand.

Several additional points should be considered when interpreting our findings. First, our estimate of IIV effectiveness is imprecise due to the small number of case patients and the low rate of IIV uptake among control patients. Low vaccination uptake is a major challenge to using a test-negative case–control design to estimate influenza VE among target groups in countries with new influenza vaccination programs. Second, some case–control studies of IIV effectiveness define control patients as those who are negative for influenza viruses but positive for other respiratory viruses to control for respiratory specimen quality and reduce case misclassification due to false-negative influenza test results.^9^,^16^ Despite extensive testing for other respiratory viruses, we were unable to use this approach because there were too few patients with respiratory specimens negative for influenza but positive for other respiratory viruses. A recent analysis comparing VE estimates based on control patients that are negative for influenza versus negative for influenza but positive for other viruses found no difference in results.^17^ Nevertheless, misallocation of case patients as control patients could have potentially led to underestimation of IIV VE in our analysis. Lastly, because this analysis was based on surveillance data, the sample size was limited by influenza circulation in the study communities and our sample size did not reach our *a priori* goal estimates based on an assumption of 50% VE. Inadequate sample size increases the risk for type II error (i.e., concluding the absence of effectiveness when the vaccine is effective) but should not have affected the validity of our estimates which reached statistical significance.

Our findings suggest that the 2010 and 2011 Southern Hemisphere IIVs were moderately effective against hospitalization with influenza-associated ARI among persons aged ≥50 years in rural Thailand and that VE was similar to that reported from studies of the same vaccine strain composition in high-income countries. Although our analysis shows that influenza vaccination is of benefit to persons aged ≥50 years in Thailand, influenza vaccine uptake was low in our study population. Efforts to increase the impact of the national influenza vaccination program in Thailand are warranted and should include strategies to increase vaccination coverage, such as targeted communication on the benefits of influenza vaccination and earlier vaccine delivery to target groups.
